# A phenomenological and quantitative view on the degradation of positive electrodes from spent lithium-ion batteries in humid atmosphere

**DOI:** 10.1038/s41598-023-32688-0

**Published:** 2023-04-06

**Authors:** Thomas Langner, Tim Sieber, Anja Rietig, Virginia Merk, Lutz Pfeifer, Jörg Acker

**Affiliations:** 1 Department of Physical Chemistry, Brandenburg University of Technology Cottbus – Senftenberg, 01968 Senftenberg, Germany; 2 LTB Lasertechnik Berlin GmbH, 12489 Berlin, Germany

**Keywords:** Materials science, Chemistry, Analytical chemistry, Chemical engineering, Energy, Inorganic chemistry, Materials chemistry, Process chemistry, Surface chemistry

## Abstract

The present study deals with the phenomenological observation of the corrosion of the positive electrode foil of lithium-ion batteries containing LiNi_0.6_Co_0.2_Mn_0.2_O_2_ (NMC) as cathode material. Due to the presence of moisture, localized water accumulation is formed on the NMC surface. The water absorbed by the electrolyte reacts with the NMC under Li^+^/H^+^ exchange and the resulting pH increase leads to dissolution of the carrier foil and characteristic salt-like blooms on the NMC surface. With the increase in the relative area occupied by the holes in the aluminum foil per time, a sufficiently suitable parameter was found with which to quantitatively determine the extent of corrosion. The degree of degradation depends on time and ambient humidity. It was shown that functional recycling with the water jet method is no longer applicable for degraded foils, since the mechanical stability of the foils decreases as corrosion progresses. Lithium, aluminum, sulfur and oxygen were detected in the blooms using SEM–EDX and Laser-Induced-Breakdown-Spectroscopy (LIBS). The underlying NMC layer was found to contain mainly aluminum and significantly lower lithium content than the non-degraded material. SEM and Raman microscopy analyses also showed that the active material is also locally degraded and therefore no longer suitable for functional recycling.

## Introduction

### General introduction, application, materials

Lithium-ion batteries (LIBs) have become the dominant technology for storing electrical energy in recent decades. The areas of application for this technology include smartphones, laptops, tablets, tools as well as mobility applications such as electric bicycles or electric cars. LIBs are manufactured in various designs. They are available as cylindrical, prismatic or so-called pouch cells. For example, LiCoO_2_ (LCO), LiMnO_2_ (LMO), LiNi_x_Mn_y_Co_z_O_2_ (NMC), LiNi_x_Co_y_Al_z_O_2_ (NCA) or LiFePO_4_ (LFP) are used as cathode materials. Graphite and increasingly silicon or Li_4_Ti_5_O_12_ (LTO) are used as anode materials. LiPF_6_ is often used as the conducting salt in the organic electrolyte. It exhibits very high ionic conductivity and leads to passivation of the aluminum collector of the positive electrode^1^.

### Strategies for battery materials recovery

Having reached the end of their lifetime, lithium-ion batteries do not represent waste, but are an important source of raw materials for all those elements that are urgently needed for the production of new batteries. On an industrial scale, processes based on pyrometallurgical, thermal and hydrometallurgical process steps have been developed in recent years, at the end of which there is always the chemical separation of the cathode material (NMC) consisting of lithium, nickel, manganese and cobalt into the pure salts of the individual elements. However, such processes require a considerable amount of energy as well as very different reagents^2–11^.

In contrast, new approaches are coming into the focus of science that are considerably more energy and resource efficient than the processes used so far, which are referred to in the literature as direct recycling^11^ or functional recycling^12^. This method aims to recover the cathode material with the least possible use of reagents while largely retaining its morphological, physical and chemical properties, so that it can be used again directly or regenerated for the production of new battery cells^12^.

The world's first industrial implementation of the principle of functional recycling is found in the decoating of positive electrodes by a water jet process. In this process, the coating consisting of NMC, conductive carbon black and binder is gently removed from the aluminum carrier foil using a high-pressure water jet, to which various additives can optionally be mixed. This comparatively simple technology makes this process the most efficient and environmentally friendly compared to the other known industrial recycling processes^13^. A particular advantage of this process is the high material quality of the recovered NMC recyclate, so that it can be used directly as an admixture to new cathode material for the production of new positive electrodes^14^.

Whether it is possible to reuse the recovered cathode material (recyclate) depends essentially on its quality and thus on what degradation the material has already suffered in the lifetime of the cell and to what extent it is possible to avoid or at least minimize degradation processes during the individual steps of the recovery process. Degradation of the cathode material occurs, for example, during the charge/discharge cycles of the cell^15,16^. It has been demonstrated that during the cycling of NMC 111^17^, NMC 622^18^ as well as NMC 811^19^ the secondary particles show cracks. Heenan showed that 1/3 of the cracks are already induced in the material during the calendaring process^15^. Furthermore, the irreversible loss of lithium due to the thickening of the SEI^20^ leads to a lack of lithium in the active material, which results in irreversible damage and loss of power while the batteries are in operation. At very high State-of-Charge SOC, the oxygen in the lattice is reduced by the Ni^4+^ ions. The released oxygen then reacts with the EC in the electrolyte, which leads to the release of CO and CO_2_. The irreversible release of O_2_ leads to a decrease in the capacity of the NMC^21^.

The process steps during the recycling procedure also lead to degradation processes that reduce the quality of the recovered cathode material. Overdischarge of the battery, can lead to irreversible deposition of copper on the positive electrode^22–24^, which leads to a deterioration in material performance and cycle stability when the recyclate is reused^25,26^.

Functional recycling in particular involves further degradation phenomena. After discharging the battery stack and separating the cells, the individual pouch cells are opened and the electrodes are separated in air. Various degradation processes take place on the NMC when it comes into contact with air humidity. A determining factor is the ion exchange between the Li^+^ ions of the NMC and the H^+^ ions of the water, which leads to an alkaline environment on the NMC surface, resulting in numerous subsequent reactions. For example, Shkrob et al. reported the formation of Li_2_CO_3_ and LiOH species on the surface of NMC-532 after storage at 100% humidity for a period of up to 2 months. The formation of these species, in which the exposure of transition metal oxides to moisture initiates an H^+^/Li^+^ cation exchange mechanism, leads to a loss of capacitance^27^. Follow-up reactions of the degradation products are also known, for example, the reaction of the resulting LiOH with residues of the conducting salt LiPF_6_^28,29^. LiPF_6_ is hygroscopic and sensitive to hydrolysis. LiPF_6_ dissociates into LiF and PF_5_. The PF_5_ is hydrolysis-sensitive and leads to a large number of hydrolysis products, which are accompanied by a release of HF^30–33^. Furthermore, HPO_2_F_2_ has been shown to react with the organic components of the electrolyte and a variety of organophosphates can be formed^33–35^. All these surface passivations of the NMC particles consisting of Li_2_CO_3_, LiOH and LiF lead to a more difficult diffusion of Li^+^^36^.

Zhang et al. showed that there is not much difference in the lattice parameters of the mixed oxides after ageing to ambient conditions. Therefore, they concluded that exposure to moisture only affects the surface layer of Li_1+x_(Ni_1/3_Mn_1/3_Co_1/3_)_1-x_O_2_. They note that both LiOH and Li_2_CO_3_ were formed on the surface of LiNi_1/3_Mn_1/3_Co_1/3_O_2_ even with short exposure time^37^. Busa et al. found that after a storage time of 28 days at ambient conditions, lithium carbonate and lithium hydroxide formed on the particle surface of an NMC 811. They further suggested that the formation of these species lead to increased cracking and consequent increase in resistance to charge transfer^38^.

When the water jet process is applied to positive electrodes coated with NMC, a long contact time of the cathode material with the aqueous decoating medium is given, which, if the process is unfavorable, may give sufficient time for the reaction of partial exchange of Li^+^ for H^+^ ions in the aqueous medium, resulting in a significant loss of lithium in the material^12^. With unfavorable process control, degradation processes can thus be triggered, which lead to dissolution of the material, disintegration of the secondary particles or unwanted deposition of hardly soluble compounds, for example Al(OH)_3_ and LiAl(OH)_4_ on the particle surface of the cathode material^12,39^.

In addition, degradation mechanisms are also known that act on the transition metal ions in the NMC. If nickel is present in the high oxidation state Ni^3+^, which is partly the case if the lithium is completely intercalated as in untreated material, the reaction with water (moisture) leads to a reduction to the oxidation state to Ni^2+^, which means a reduction of the specific capacity^40–42^. During exposure to moisture, the reduction of Ni^3+^ to Ni^2+^ at the particle surface produces active oxygen species that combine with H_2_O and CO_2_, and with Li^+^ ions eventually lead to the formation of lithium carbonate and hydroxide species^43^.

The present study is devoted to a particular phenomenon of functional recycling of batteries with NMC as cathode material using water jet technology. The main focus is on the degradation processes that start immediately after opening the pouch cells and removing and separating the positive electrode foils in contact with air and moisture. It can be shown that with increasing storage time under ambient conditions, the decoating process becomes more and more incomplete and, in extreme cases, the entire positive electrode foil can be destroyed, so that eventually even particles of the aluminum foil can get into the recyclate.

## Results

### A phenomenological perspective

**Figure 1:**
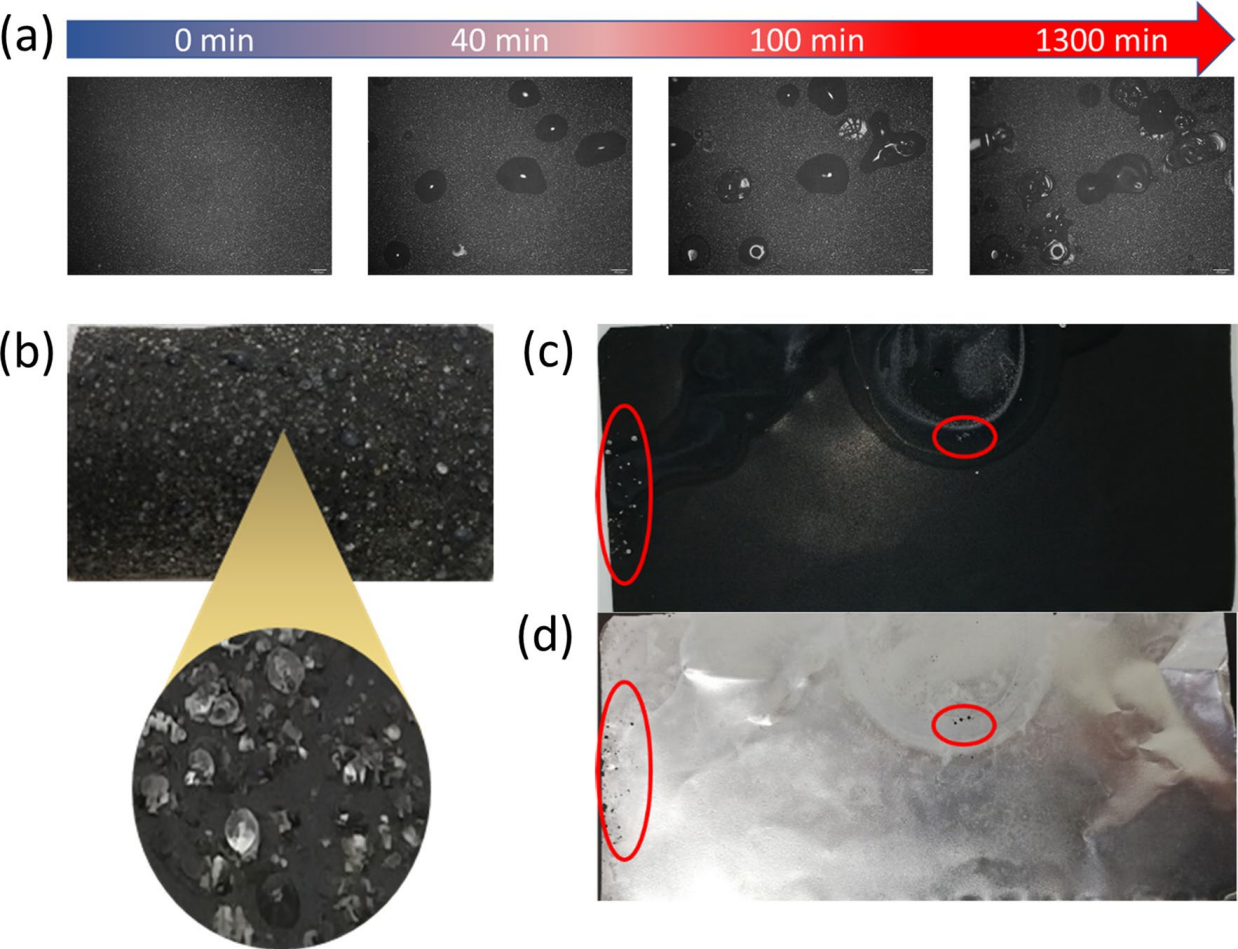
(**a**) Time sequence of the accumulation of moisture on the surface of a positive electrode; humidity of φ = 100%, 2.55 mm × 1.91 mm; (**b**) Precipitations on a positive electrode surface (removed from a used LIB) stored in air for 14 days at a humidity φ = 100%, enlarged section with a diameter of Ø ≈ 2.5 mm; (**c**) Precipitations on a virgin positive electrode (without any contact with an electrolyte) that has been wetted with an electrolyte only in the areas outlined in red, (**d**) holes are detectable in the decoated aluminum substrate foil only where the precipitations has been formed as a result of contact with the electrolyte.

If positive electrode foils are stored in air immediately after their removal from the pouch cell, moisture collects at seemingly random locations on the positive electrode surface due to the hygroscopicity of the electrolyte as well as the positive electrodes (Supplementary Video S1 online, Fig. 1a). At the beginning, the fluid accumulations are not visible at this scale, but only by microscopy under strong illumination. In the further course, small droplets become visible on the surface and after a further period of time white dots or larger crystalline blooms. In the following, storage in air will be referred to synonymously as ageing.

Positive electrodes from used LiBs that have been stored in air for several days develop white, salt-like precipitates on the surface of the NMC coating, which can reach typical diameters between 100 and 500 µm (Fig. 1b). If the NMC coating is removed from a positive electrode that is already showing the first salt-like precipitates, a large number of small holes become visible in the exposed aluminum substrate foil. Positive electrodes that have never been in contact with a typical liquid battery electrolyte do not show any of the typical symptoms of aging when stored in air for weeks. If such a virgin positive electrode, which has never been in contact with an electrolyte, is exposed in some spots with a liquid electrolyte taken from a spent cell of the same type, the salt-like precipitates will also develop in the course of a day, and the underlying aluminum foil will show the typical holes at exactly these spots after decoating (Fig. 1c,d).

**Figure 2:**
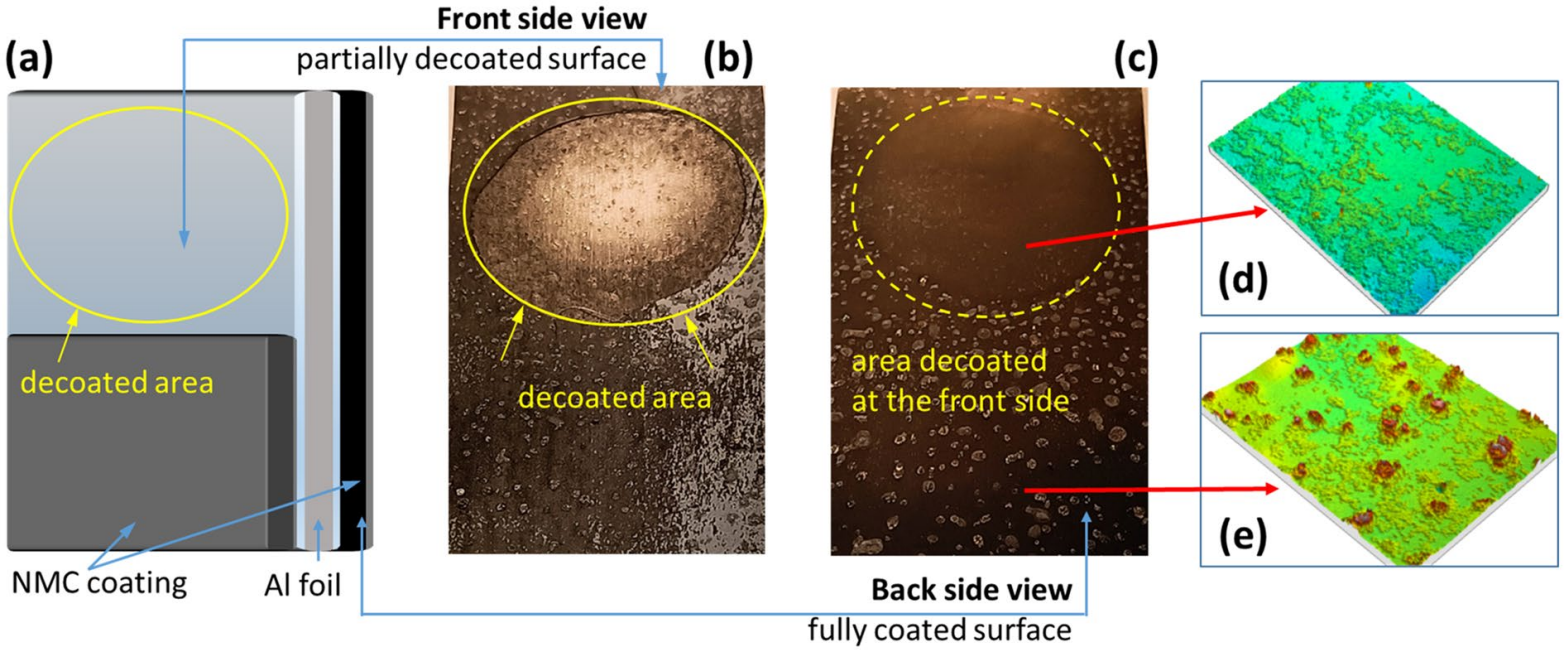
(**a**) Schematic illustration of the partially decoated positive electrode; (**b**) optical image of the partially decoated front side and of (**c**) the fully coated back side after aging in air humidity of φ = 97% for 18 h; confocal microscope images electrode surface taken (**d**) from the apparently non-corroded area with decoating on the surrounding side and (**e**) the surrounding corroded electrode surface formed in areas with NMC coating on both sides; light–dark patterning seen inside the yellow circle in the optical image (**b**) and (**c**) are only due to light reflection.

For a more detailed investigation of the degradation effects, a positive electrode consisting of an aluminum substrate foil (thickness 12 µm) coated on both sides with approx. 65 µm NMC was partially stripped of the NMC layer on one side by means of an adhesive strip and then stored for about 18 h in an atmosphere with 97% humidity. Figure 2a shows a schematic diagram of the chosen experimental setup. The partially stripped area of the positive electrode foil is formally referred as the front side (Fig. 2b), while the area still fully coated is referred to as the back side (Fig. 2c).

After the aging process had been completed, the following picture emerged: The aluminum substrate foil exposed on the front side shows the previously described fine perforation and also exhibits a large number of white and salt-like precipitates (Fig. 2b). The adjacent, still present NMC coating shows a nearly similar large abundance of these white precipitates (Fig. 2b). Surprising is the image from the back of the positive electrode. The optical image in Fig. 2c illustrates that only the areas with intact NMC coating on both sides show the white, salt-like deposits on the surface. In contrast, the area of the reverse side, which was decoated on one side before storage in air on the front side, shows absolutely no traces of degradation and also no visual nor haptic changes can be detected. This is illustrated in Fig. 2d and e by confocal microscopy images of the surface.

Based on Fig. 2d and e, this different behavior can be quantified using the surface area ratio, Sdr, which covers all features of a surface topography as ratio between the total (folded) surface area, A_S_ and the geometric (projected) surface area, A_P_^44–46^. Starting with a common geometric surface area of 12.4 mm^2^ (A_P_), the positive electrode surface has a folded surface area of 13.2 mm^2^ (A_S_) resulting in a Sdr of 1.064 when the backside is decoated and the aluminum substrate surface is exposed. In the area with NMC coating on both sides and their clearly visible deposits, almost a doubling of the folded surface is observed measuring A_S_ = 20.1 mm^2^ resulting in a Sdr of 1.621. The small irregularities on the surfaces with 5 to 7 µm in height in Fig. 2d and e as well as the whitish haze across the entire positive electrode foil (Fig. 2c) originate from adherent residues of the ceramic coating of the separator foil, which are of no relevance for the present investigations. In addition to the salt-like precipitations, the increasing roughness of the surface of the NMC coating is also attributable to an increase in the volume of the crystallizing salts in the pores of the coating. Only in the case of the single-sided decoated positive electrode the pores of the NMC coating constitute a higher diffusion resistance, so that the salt-containing liquid passes through the holes to the aluminum/air interface with lower transport resistance and crystallizes there. As a result, the surface of the NMC coating shows no salt-like precipitations and also no roughening of the surface, because crystallization and volume increase occur on the decoated side.

### Time-dependent degradation in air and under argon

The following investigation is focused on the question of which of the components of the electrolyte are responsible for the absorption of atmospheric moisture. In particular, it is important to clarify whether the hydrolysis-sensitive conductive salt components, for example LiPF_6_, or the hygroscopic volatile organic electrolyte components exert the determining influence. Furthermore, it should be noted that the active materials also have a hygroscopic character.

For this purpose, a parallel procedure was chosen by opening the pouch cell of an end-of-life LIB in a glove-box under argon atmosphere and dividing the electrode stack. From a portion of the stack, the positive electrodes were separated from the separator foils and the negative electrodes in ambient air (relative humidity φ ≈ 55%) to evaporate the volatile electrolyte components in air from the beginning. The second portion of the positive electrodes was separated from the negative electrodes and separator under argon and the electrolyte components were first evaporated under argon. Then the positive electrodes were stored under vacuum for 24 h to facilitate most of the volatilization of the solvents present in the pores before the positive electrodes were exposed to ambient air (relative humidity φ ≈ 55%).

**Figure 3:**
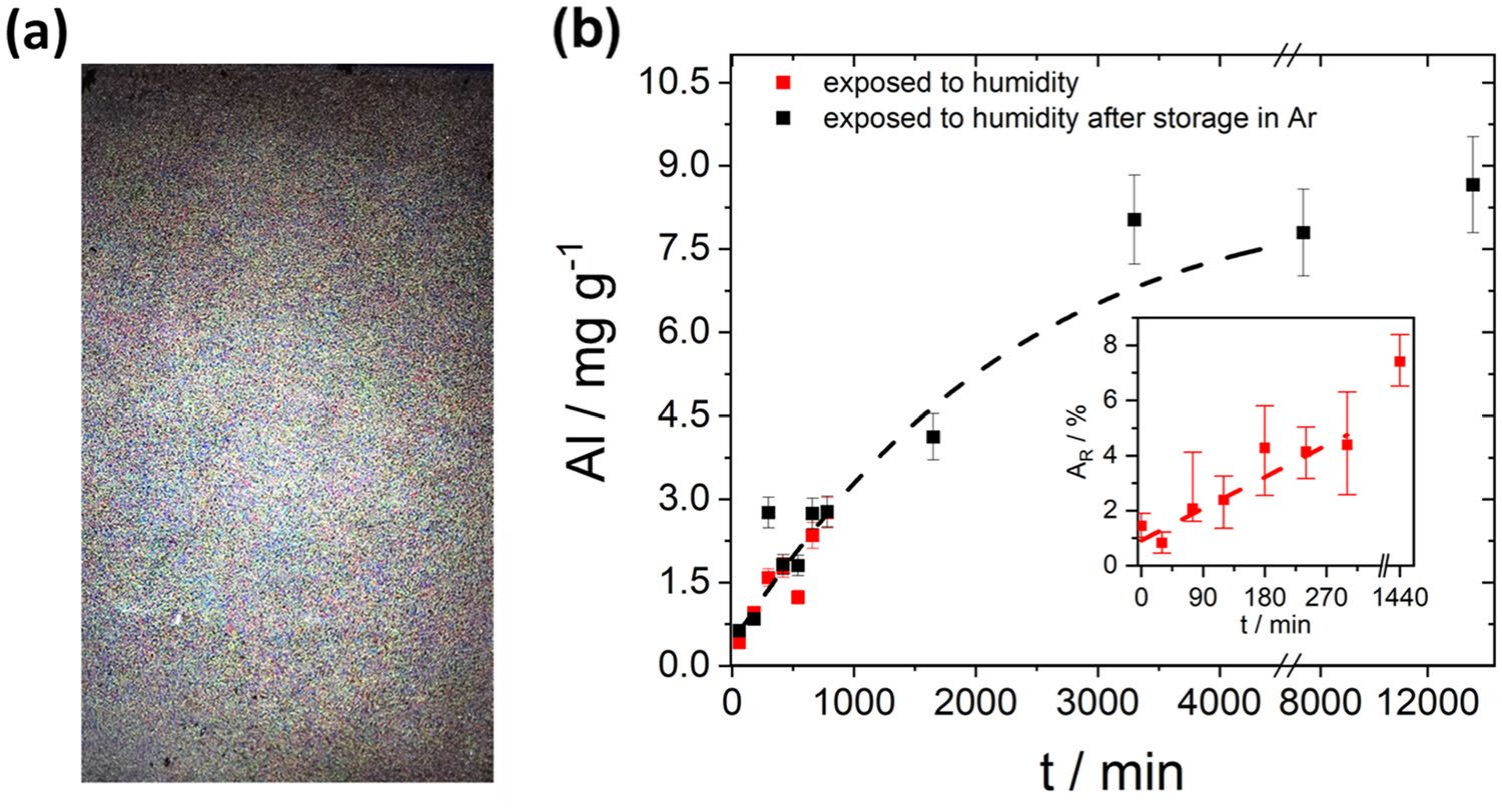
(**a**) after 24 h in air stored, decoated carrier foil (transilluminated); (**b**) Aluminum content of the cathode material as a function of exposure time at φ ≈ 55%, Inset: relative area of the holes of the foils during storage at ambient air humidity of φ ≈ 55% as a function of time; the dashed lines are guide-to-the-eyes.

Beginning with the time of contact with ambient air, individual positive electrodes were decoated at defined intervals using a water jet. The positive electrodes, whose volatile electrolyte components evaporated in ambient air, were increasingly difficult to decoat over time (10–300 min), since the adhesion of the positive electrode coating to the substrate foil increases. In parallel, the decoated aluminum substrate foils showed a steady increase in the number of small holes, which was already visually recognizable (Fig. 3a).

The extent to which the formation of holes in an aluminum substrate can reach is shown as an example in Fig. 3a for a positive electrode that was decoated after a treatment time of 24 h at a humidity of φ ≈ 55%. To make the resulting holes visible, the aluminum foil was placed on a white luminescent LCD display so that the holes appear as bright dots. It must be emphasized that the holes are evenly distributed over the entire foil; the optical image results in optical aberration, indicating that there are more holes in the center of the foil (at the focus of the camera's optical lens) than at the edges.

For the positive electrodes, where the volatile electrolyte components were evaporated under argon and in vacuum, no change in the decoating behavior was observed after subsequent storage in ambient air for up to 180 min. Thereafter, adhesion increased steadily, so that for a total storage time of 24 h, complete decoating was still possible, albeit with a more intensive rinsing process.

The corrosion of the aluminum foil can be clarified on the basis of two investigations. The most reliable statements are those resulting from time-dependent determination of the dissolved aluminum in the active material. Figure 3b shows that the aluminum content in the active material increases continuously without any difference between drying in air or drying in argon as soon as the positive electrodes are exposed to ambient air. After about 3350 min, the aluminum content approaches saturation. Thus, the degradation has a self-limiting character. The fact that the corrosion of the positive electrodes stored under argon proceeds at almost the same rate as the positive electrodes directly exposed to air suggests that it is the non-volatile conductive salt residues that initiate the corrosion of the aluminum through their hygroscopic properties.

The second method of investigation is based on quantifying the area of the holes formed in the aluminum foil. Here, the relative ratio between the area of the holes formed as time progresses and the total area of the aluminum foil (A_R_) is determined by microscopy. The inset in Fig. 3b shows a linear increase in the relative hole area with considerable dispersion. It should be noted that the quantification of corrosion via the temporal increase in the geometric area of the holes has only limited significance, since the simultaneously occurring process of areal corrosion, which leads to a decrease in the foil thickness, is not taken into account.

### Humidity-dependent degradation

**Figure 4:**
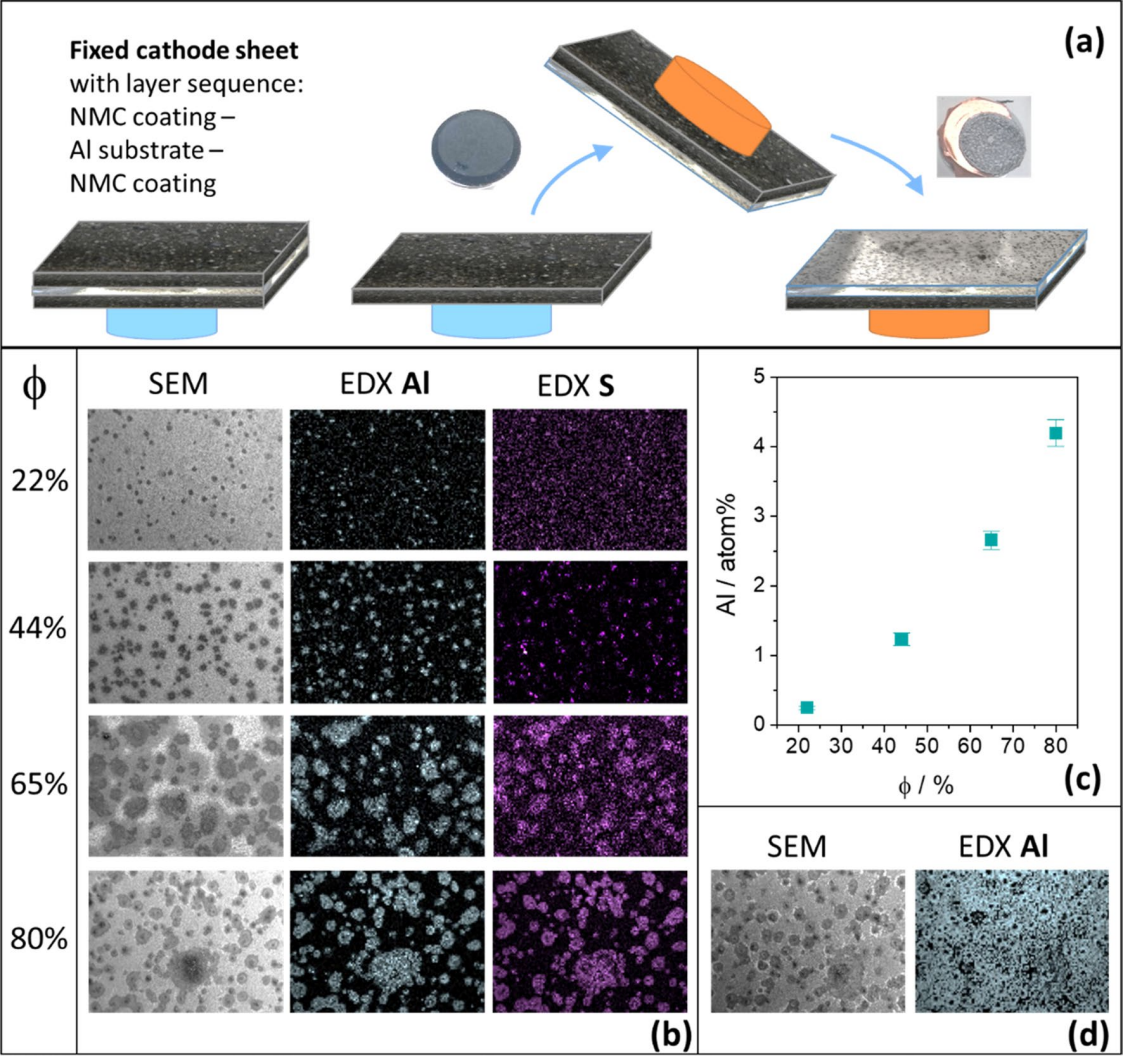
(**a**) Schematic illustration of the separation of the layers of the positive electrode foil in preparation for analysis after ageing; (**b**) SEM images and SEM–EDX analyses of a fixed section of 5.5 mm × 4 mm on the NMC coating with substrate contact area as a function of air humidity; (**c**) SEM–EDX analysis of aluminum content on the substrate contact surface of the NMC coating with increasing air humidity; (**d**) SEM image and SEM–EDX analysis (5.5 mm × 4 mm section) of the decoated aluminum substrate after 18 h at 80% air humidity.

The dependence of the degradation of positive electrodes from end-of-life LIBs on humidity was investigated. The foils used for these experiments were separated after opening the battery in air and then transferred to an argon atmosphere. Pieces of these positive electrodes were glued to SEM sample plates and exposed upside down to an atmosphere of defined humidity for a total of 18 h (Fig. 4a). After the 18 h aging process, the positive electrode foils were separated according to the scheme in Fig. 4a by sticking them on another sample plate and tearing them off jerkily in such a way that a separation occurred at the interface between the NMC coating and the aluminum foil, so that both the surface of the aluminum foil and the contact area of the NMC coating to the aluminum foil were available for further investigations.

The SEM images as well as the corresponding SEM–EDX mappings in Fig. 4b make clear that the aluminum content in the NMC coating increases more and more with increasing humidity. For a humidity of 97%, it was no longer possible to perform such an analysis, as the degradation of the aluminum foil made it impossible to separate the layers. If one plots the aluminum content determined by SEM–EDX in the examination area as a function of the air humidity (Fig. 4c), a direct, disproportional correlation is found. If one also looks at the exposed aluminum substrate, it becomes clear from the SEM–EDX mapping that at 80% humidity (Fig. 4d) the aluminum foil is interspersed with holes in such a way that the underlying NMC coating becomes recognizable and detectable by means of EDX. Furthermore, it is evident that the aluminum is associated with the sulphur (Fig. 4b). The SEM–EDX analyses show that up to 65% humidity there is an increasing content of fluorine associated with the aluminum and sulphur. At 80% humidity, the fluorine content on the NMC layer is lower and there is no clear association with other elements. The complete SEM–EDX analyses are shown in Supplementary Figure S1 online and Supplementary Table S1 online.

**Figure 5:**
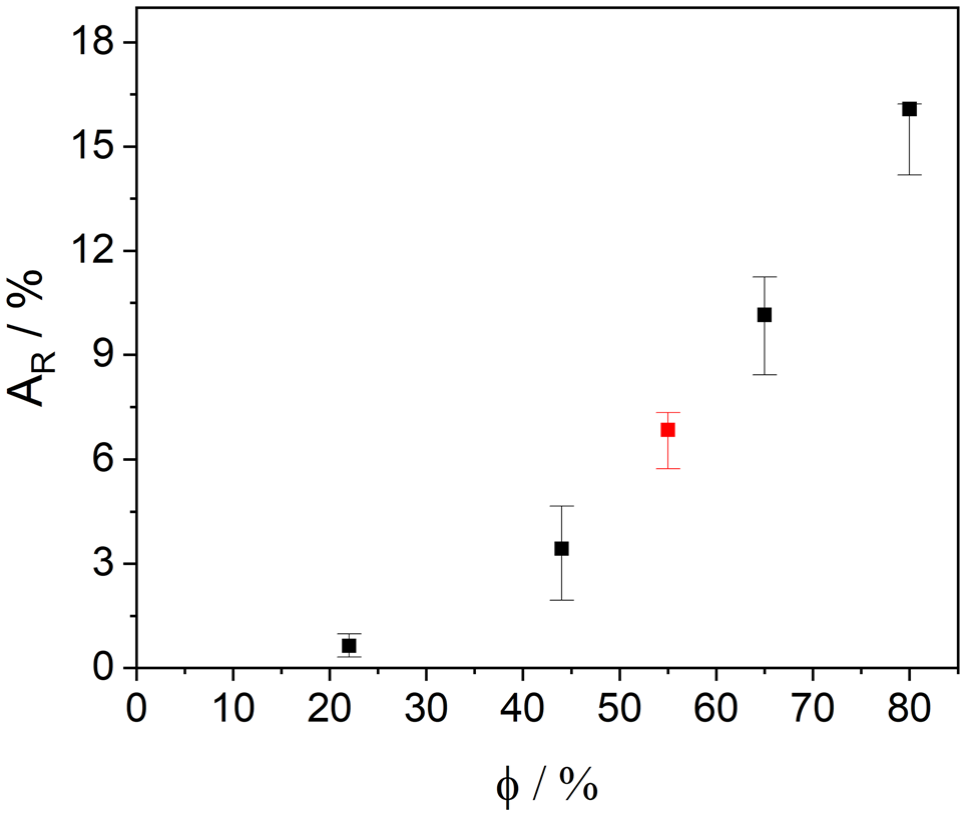
Correlation of the relative area of the holes during 18 h storage (black dots) at different degree of humidity, red dot at a humidity of φ = 55% end point from Figure 3b interpolated to 18 h.

With the help of the experimental setup just described, it is possible to analyze the contact surfaces of aluminum foil and NMC coating in isolation. However, this experimental set-up carries the risk of influencing the processes taking place because the moisture can only reach the positive electrode from one side, as the back of the positive electrode is practically glued airtight to the sample plate. Therefore, the functional relationship between the duration of the access of humidity on both sides and the resulting perforated area of the degraded aluminum foil was determined with a modified experimental set-up. Positive electrodes were positioned in a beaker, which served as an air chamber, so that the air humidity could reach both sides of the positive electrodes unhindered. After the 18-h ageing process, the positive electrodes were completely decoated using a water jet and the total area of the holes in the aluminum foils was examined using confocal microscopy. When the foil was transilluminated, it was already clear that the first local perforations of the foil had taken place at 22% humidity, although the NMC coating appeared visually intact and unchanged before its removal. Even at 44% humidity, the NMC coating still showed no visually recognizable salt-like bloom. The removal of the coating worked without any problems. However, there was a perforation of the carrier foil over a large area. Similarly, upstream tests showed that corrosion had already taken place on the contact surface of the NMC to the aluminum (Fig. 4b,c) although the effects on the positive electrode surface were not yet visible. After 18 h of ageing under 65% humidity, the first bloom and droplets of liquid finally appeared on the surface and the first difficulties in decoating the positive electrode became apparent and the aluminum foil was already perforated over a large area. After 18 h of exposure under 80% humidity, the decoating by means of a water jet failed, as the punctual impact of the water jet led to the complete destruction of the foil. In order to be able to make a quantitative statement about the degree of destruction, a foil aged in this way was decoated by thermal decomposition of the adhesive binder and careful "brushing off". Under the influence of 97% humidity, the degree of degradation of the aluminum substrate was finally so high that no aluminum foil could be separated even after thermal decomposition of the binder. Figure 5 shows the result of the confocal microscopic examination of the perforated area per analyzed 24 cm^2^ aluminum substrate with increasing air humidity.

Thereby it can be seen that with increasing humidity, a greater extent of degradation in the form of holes can be observed. The end point from Fig. 3b fits into the results of this test seamlessly (Fig. 5, red dot). Furthermore, the decoating deteriorates with increasing exposure to air humidity.

Analogous to the transfer of aluminum to the NMC coating analyzed by SEM–EDX (Fig. 4c), the degradation of the aluminum substrate, measured by the number of holes, also shows a direct correlation with increasing air humidity content (Fig. 5).

### Quantitative observations: the salt-like bloom

**Figure 6:**
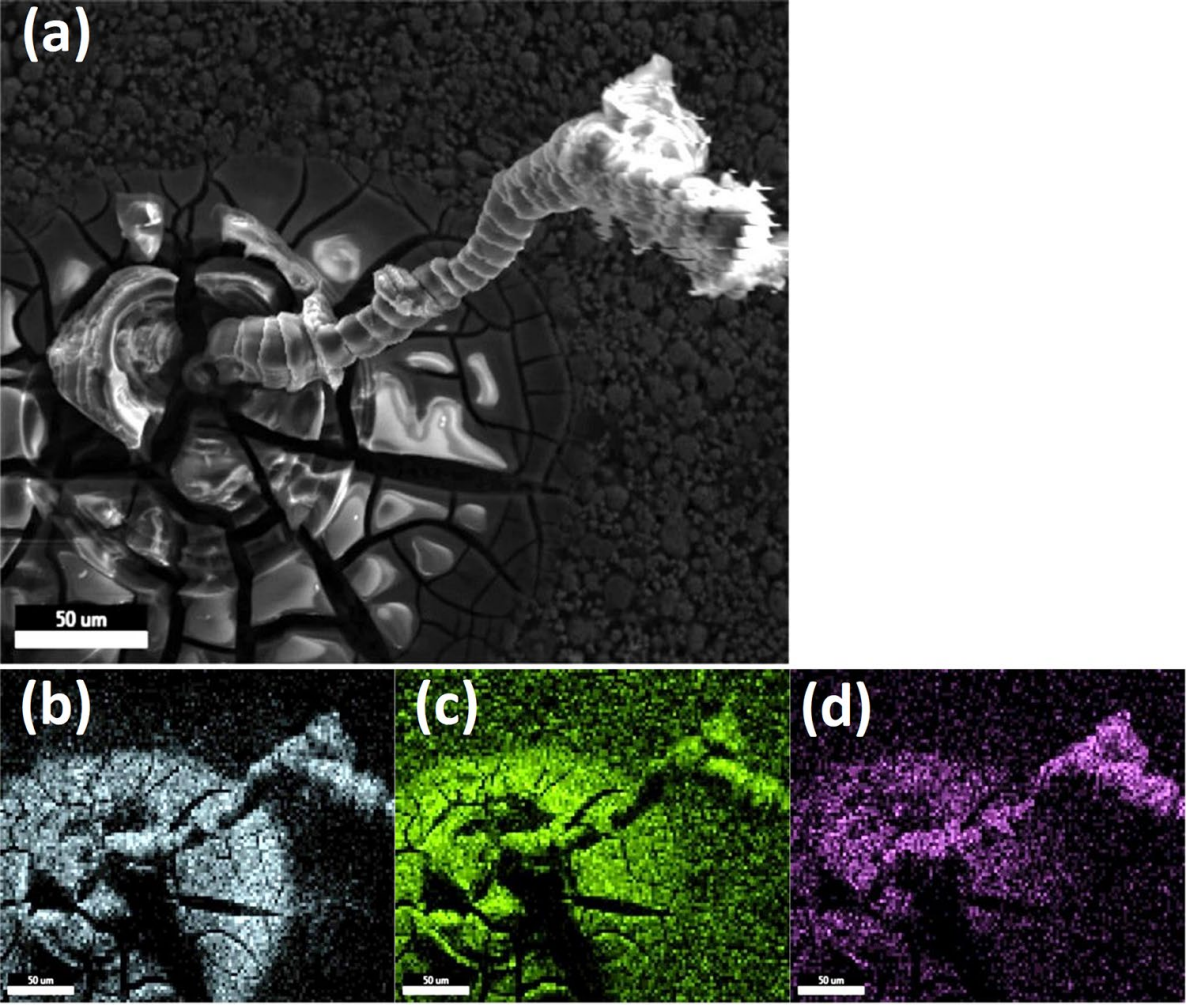
(**a**) SEM image of the stalactite-like crystallite; EDX element mapping for (**b**) aluminum, (**c**) oxygen, (**d**) sulphur of the crystallite; humidity φ = 80% for t = 18 h.

The findings suggest that the aluminum foil dissolves in the course of degradation in a locally formed alkaline medium. Dissolved aluminum salts are transported into the NMC layer, and crystallization occurs within and on the NMC coating. Aluminum, sulphur and oxygen were detected in the bloom. A reliable chemical characterization of these precipitates is difficult in the presence of the NMC coating and the aluminum foil. Therefore, an attempt was made to isolate this material. For this purpose, pieces of positive electrodes were placed on top of the humidification apparatus in high humidity of 80% for a period of 18 h. The high humidity ensured that the dissolved salts did not crystallize directly on the surface of the coating, but caused its steady transport by gravity. As a result, stalactite-like crystallizations were obtained on the surface of the NMC coating (Fig. 6a), which could be analyzed by SEM–EDX. The analyses revealed that the crystallite consists predominantly of aluminum (Fig. 6b) and oxygen (Fig. 6c), and it was also found that considerable amounts of sulphur (Fig. 6d) were evenly distributed in the solid.

Al(OH)_3_ should be a main component of the crystallite. A presence of LiAl(OH)_4_^12^ or [LiAl_2_](OH)_6_·H_2_O^47^ would also be plausible, but lithium cannot be detected by EDX. The detection of sulphur, however, is an unexpected finding. It can be assumed that the source of the sulphur is the electrolyte used and contains at least one sulphur-containing conducting salt. By means of ICP-OES and ion chromatographic analysis of the electrolyte from the pouch cells examined, Sulphur (0.15 mol L^−1^), phosphorus (1.08 mol L^−1^), lithium (1.15 mol L^−1^) and fluorine were detected. The conducting salt components, fluorine and phosphorus, were not detected in the blooms. Raman spectroscopic analyses of the blooms showed partial evidence of sulphate (υ_S–O_ = 981 cm^−1^)^48^.

The stoichiometric ratio between aluminum, oxygen and sulphur within the salt-like precipitate determined from the EDX images is n(Al):n(O):n(S) = (2.1 ± 0.1): (4.8 ± 0.4) : (1.0 ± 0.1). A plausible interpretation of the present element ratios is not possible with the data available so far. However, the fact that fluorine was only found at the interface to the aluminum indicates that due to the better solubility, the sulphur-containing compounds are further driven out of the active material and thus the fluorine-containing ones are no longer detectable in the bloom on the NMC surface. Thus, predominantly sulphur-containing compounds are detected.

In a parallel experiment, 2 freshly taken positive electrodes were intensively rinsed with ethanol to remove the electrolyte used in the battery from the electrodes. Subsequently, a commercial standard electrolyte (LP30, Sigma Aldrich) was added to one electrode and both electrodes were stored for a period of 24 h at a relative humidity of 62%. The measured hole area AR of the electrode treated with the commercial electrolyte shows no significant difference from the AR determined in experiments where the sulfur-containing component of the electrolyte was present. If the electrolyte is removed from the electrode, as in the parallel experiment, there are effectively no holes detectable in the carrier foil after 24 h. Thus, the extent of degradation of the positive electrode at a given humidity is subject to the presence of the electrolyte (Supplementary Figure S2 online). Whether the sulfur-containing component is actively involved in the dissolution process or merely dissolves in the alkaline medium is not clarified in this study.

In order to obtain information about the chemical composition of the crystallites within the NMC coating, selected samples were analyzed using laser-induced breakdown spectroscopy (LIBS), which is able to detect lithium. Using a laser power of 3 mJ and a grid of 5 × 5 measuring points an area of 0.2 × 0.2 mm^2^ was sampled. In a preliminary investigation on a virgin positive electrode, the ablation depth and ablation volume for successive ablation steps were determined by confocal microscopy. In the 1st ablation step, the ablation volume is approximately 7.1 × 10^–4^ mm^3^. However, it should be noted that the error in the determination is greatest for smaller ablation volumes. From the 2nd ablation step onwards, at a mean ablation depth of 10 µm, the ablation volume is (4.8 ± 0.2) × 10^–4^ mm^3^ and remains constant for the further ablation steps. This means that with the 6th ablation step the aluminum substrate is reached.

**Figure 7:**
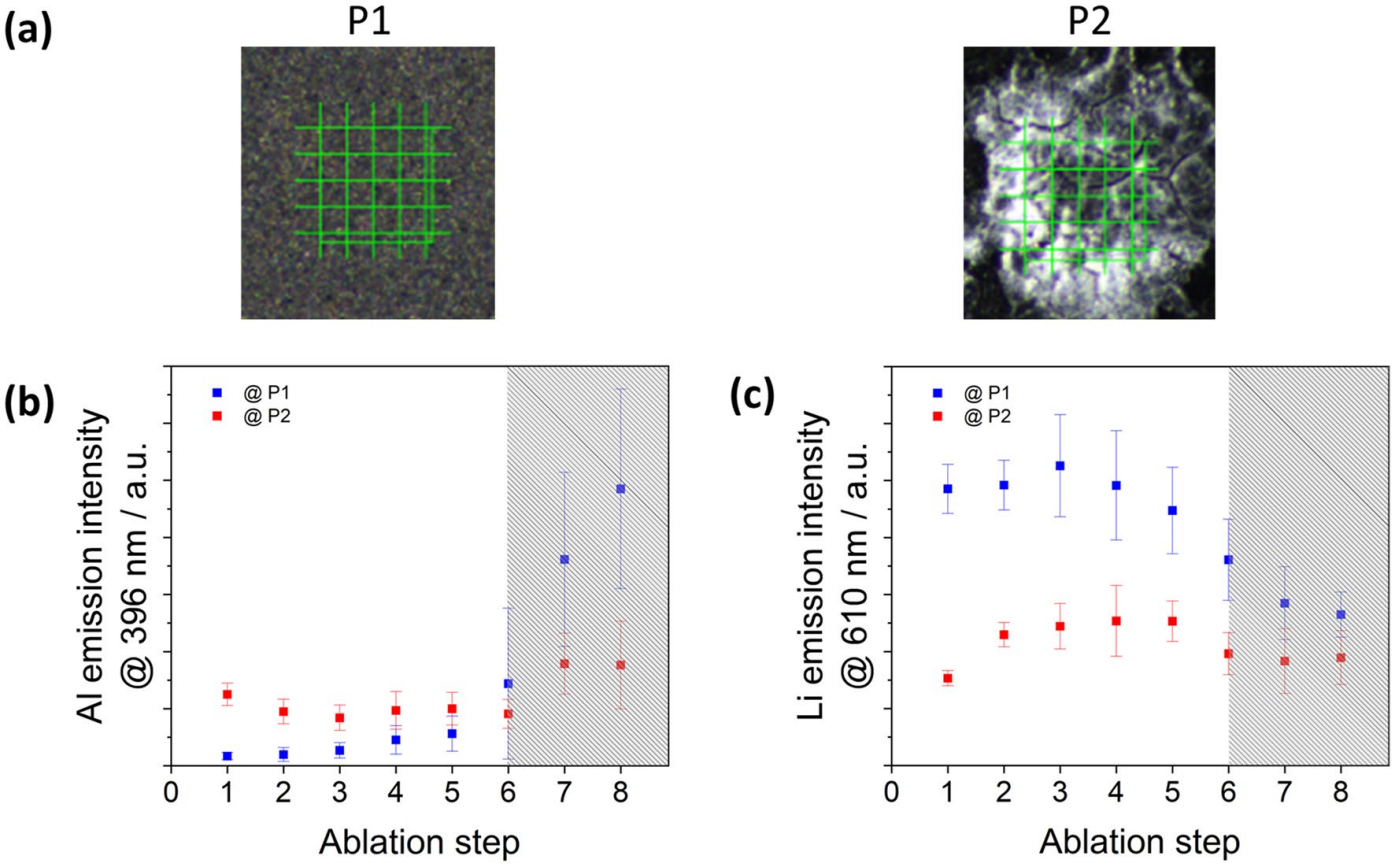
(**a**) P1 non-degraded NMC surface; P2 bloom due to degradation by air humidity of φ = 97% for t = 18 h; (**b**) emission intensity of the aluminum line at 396.15 nm (hatched: carrier foil); (**c**) background-corrected emission intensity of the lithium line at 610.36 nm (hatched: carrier foil); points represent the mean values and the error bars the standard deviation.

For the investigation of the crystallite, a positive electrode foil was examined that was stored for 18 h at 97% humidity. Two different areas of the NMC coating were investigated. One where no crystallization or bloom was detected (P1, Fig. 7a) and one with a salt-like bloom on the surface (P2, Fig. 7a). Figure 7b shows the intensity of the emission line of aluminum at 396.15 nm on the two areas P1 and P2 for different ablation steps. For P1 the emission intensities of the ablation steps 1 to 5 merely represent the fluctuation of the background signal, while the intensity increases abruptly from the 6th ablation step until the aluminum substrate is finally detected in the 7th and 8th ablation step (blue squares in Fig. 7b). For P2 already in the first ablation step, a considerably higher aluminum content is detectable, which is almost equally distributed throughout the entire NMC coating up to ablation step 6. With the 7th ablation step, the aluminum foil should be reached, but the emission intensity of aluminum increases only slightly even at depths of > 65 µm and does not reach the values achieved for P1 (red squares in Fig. 7b). This loss of intensity indicates a significant destruction of the aluminum foil directly below the salt-like bloom.

Figure 7c compares the intensities of the lithium line as a function of ablation depth on a visually undisturbed area of the NMC coating at P1 and the salt-like bloom at P2. Without exception, the undisturbed NMC coating at P1 shows a significantly higher lithium signal over the entire coating thickness. After the 6th ablation step, the NMC coating at P1 is completely penetrated and the aluminum foil is reached. The lithium emission intensity still present can be attributed here to edge effects of the ablation crater. In parallel, the difference in emission intensities of the measurements of point 1 and point 2 are recorded for nickel (231.60 nm), cobalt (228.62 nm) and manganese (257.61 nm) and also for iron (240.49 nm) and phosphorus (213.62 nm) within the NMC coating. There are no significant differences between the measurements of point 1 and point 2 (Supplementary Figure S3 online). P2, on the other hand, shows significantly lower emission intensity for lithium, indicating that the lithium is deintercalated from the NMC present by the impact of moisture. Of significance, the 1st ablation for the salt-like compound on P2 shows signals only for aluminum and lithium. This supports the hypothesis that the blooms and crystallites contain at least some lithium content that was removed from the NMC of the coating and led to its degradation.

**Figure 8:**
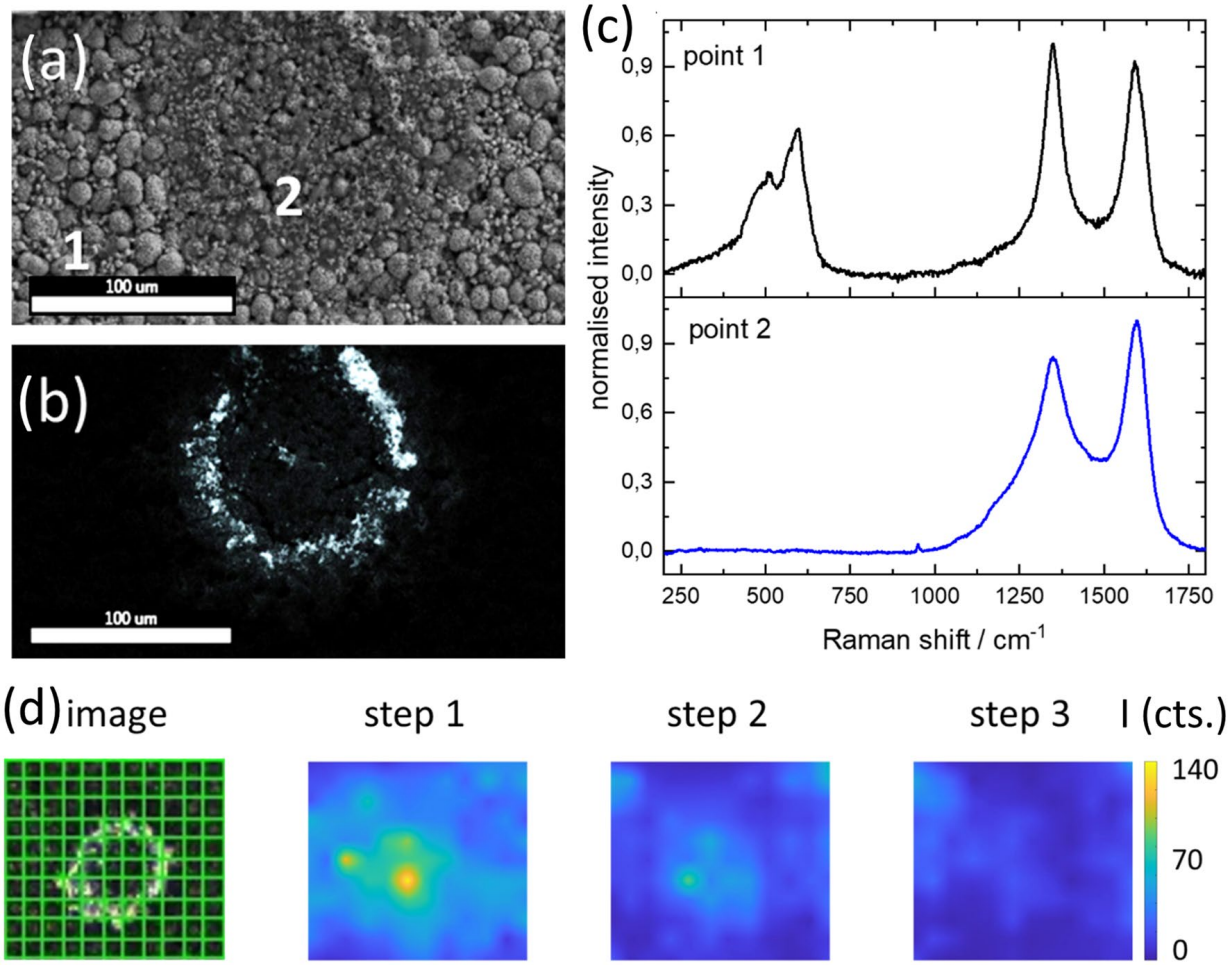
(**a**) SEM image of a local degradation NMC backside (contact surface aluminum-NMC) 1) non-degraded area, (2) degraded area; (**b**) EDX mapping of aluminum; (**c**) Raman spectra of point 1 and 2 d) LIBS analysis of a crater of the NMC backside for aluminum @ 396.15 nm, step means ablation step, size of mapped area 200 µm × 220 µm, the color bar displays the color associated with the absolute intensity (I in cts.)

In the course of the investigations, it was possible to completely separate the contact surface NMC coating—aluminum foil of a positive electrode foil with salt-like blooms by removing it with an adhesive strip. Figure 8a shows the SEM image and Fig. 8b an EDX mapping for aluminum of the NMC coating that was directly on the aluminum foil. A crater-shaped structure can be seen. Inside these craters (Fig. 8a, area 2), the positive electrode material seems to have undergone strong morphological degradation, so that secondary particles are no longer visible. Around the crater (Fig. 8a, area 1), on the other hand, there is no evidence of morphological degradation. What Fig. 8a is not able to show becomes visible in EDX mapping for aluminum or in a light image. The inner area 2 is separated from the outer area 2 by a thin rim of an aluminum-containing deposit.

The Raman microscopic analysis of both areas shows that the degradation in area 2 is not only morphologically but also chemically advanced to a high degree. Thus, the Raman spectra in area 1 without degradation (Fig. 8c, point 1) show vibrations typical for the NMC between 300 and 700 cm^−1^^12^ and the D and G bands of the conductive graphite (1350 cm^−1^, 1590 cm^−1^) ^49^. Inside the crater, however, the vibrations typical for the NMC are completely absent (Fig. 8a,c, point 2), which indicates the destruction of the layered oxide structure in addition to the proven destruction of the secondary particles.

An example of crystallization containing aluminum that has not yet reached the surface of the positive electrode coating is shown in Fig. 8d. The area shown in Fig. 8d was mapped with LIBS with a step size of 20 µm and the intensity of the aluminum line at 396.15 nm was determined for three consecutive ablation steps. The results show that at different ablation steps (Fig. 8d) a decrease in the aluminum signal is observed with each new ablation step, which most probably correspond to a concentration gradient of aluminum in the NMC coating. Aluminum is considerably enriched at the aluminum/NMC coating interface in the area of the crater and was transported from there through the NMC coating towards the surface. The fact that the aluminum is no longer clearly detectable in the third ablation step suggests that it has not yet reached the surface at this point and thus no bloom has yet occurred on the surface.

As Fig. 8d shows, the measurement grid includes both the circular, aluminum-containing area and its surroundings, in which no aluminum was detected and the NMC coating was obviously not subject to any recognizable degradation. The emission intensities of the transition metals nickel, cobalt and manganese do not show any systematic deviations in the entire area and for each ablation step, which is why these elements can be considered as equally distributed in the measurement area (Supplementary Figure S4 online). Accordingly, the transition metals also exist within the crater of their original distribution and magnitude, but the structure for lithium intercalation is largely destroyed on the basis of the Raman investigations (Fig. 8c) however, no systematic distribution during the individual ablation steps could be detected for lithium.

## Discussion

Positive electrode foils from end-of-life LIB's undergo immediate degradation when they come in direct contact with air, with the rate of degradation increasing with increasing humidity. The first step in this degradation is the rapid absorption of water from the ambient air by the hygroscopic electrolyte components. The accumulation of water is probably caused by the non-volatile electrolyte components, primarily the residues of the conducting salts adhering to the positive electrodes, which hydrolyze under water absorption and partially decompose. Regardless of whether the volatile components were evaporated in air or argon, the rate of aluminum uptake by the NMC-coating is identical.

Corrosion of the positive electrode foil is most clearly visible in the form of white, crystalline precipitates, which form on the positive electrode surface with increasing numbers and sizes over time. At the same time, the positive electrode loses mechanical stability until it finally breaks apart under slight stress and disintegrates into flake-like pieces.

The chemical reason for the corrosion is the reaction of the water with the NMC resulting in an alkaline medium^12,39^. A chemical reaction scheme for the following processes is shown in Supplementary Figure S5 online. The alkaline medium diffuses through pores in the NMC to the aluminum carrier foil, where it leads to local dissolution of the aluminum. By diffusion in the liquid-filled pore, the soluble aluminum compound can reach the surface of the positive electrode, where the white, crystalline bloom is formed by evaporation. The material transport takes place along the lowest transport resistance which could be shown by lowering the transport resistance due to one-sided decoating. The SEM–EDX as well as the LIBS analyses show that the crystalline precipitates probably consist mainly of Al(OH)_3_, LiAl(OH)_4_ and/or [LiAl_2_](OH)_6_·H_2_O. Furthermore, sulphate could be detected, which results from the alkaline hydrolysis of sulphur-containing conducting salt compounds, while fluorine and phosphorus, which were also contained in the liquid electrolyte as the conducting salt LiPF_6_, could not be detected. Our results suggest that the corrosion is self-limiting and is only determined by the value of the air humidity meaning the higher the air humidity, the more aluminum is dissolved.

If the corrosion has progressed so far that holes have already formed in the aluminum carrier foil, then the increase in the hole area per time is a sufficiently suitable parameter for quantitatively recording the degree of corrosion. However, the exact correlation to the dissolved amount of aluminum is not reflected because the local decrease in foil thickness without hole formation is not recorded.

LIBS analyses showed that lithium and aluminum coexist in the crystalline precipitates on the positive electrode surface. The LIBS depth profiles show that aluminum is present throughout the whole investigated depth while the intensity of the lithium line is clearly below that of a non-degraded NMC layer. From that we conclude that the NMC was so massively damaged by the moisture that the majority of the lithium was removed from the NMC and preferentially crystallized on the surface together with the dissolved aluminum. This finding is strengthened by the results of the SEM and Raman spectroscopic analyses of the interface between the NMC coating and the aluminum carrier foil. Outside the degradation zone formed by the ring-shaped accumulation of aluminum salts, the NMC secondary particles appear completely intact and the Raman spectra shows the typical NMC bands. Within the ring-shaped degradation zone, the NMC secondary particles are hardly recognizable anymore and the Raman spectrum is dominated by the bands of the conductive carbon black, while typical NMC bands are completely absent. It is thus concluded that the locally absorbed humidity and alkaline medium formed in the first reaction with the NMC leads to further degradation of the NMC particles, even if this process occurs more slowly than in the acidic medium^12^.

The investigations carried out reveal the consequences for possible recycling processes. The process duration between the opening of the pouch cells and the first contact of the positive electrodes with air becomes the determining parameter for the subsequent recycling processes. Due to the successive dissolution of the aluminum carrier foil, the positive electrode increasingly loses mechanical stability. Any process step that exerts a mechanical stress on the positive electrodes can therefore lead to deformations, the formation of holes or the complete rupture of the foils. A material degraded in this way can practically no longer be separated into NMC and aluminum using mechanical processes. Likewise, the fragments created during tearing can lead to unwanted contamination of other material fractions in the recycling process. As the corrosion of the aluminum foil progresses and the aluminum salts begin to crystallize in the pores of the NMC coating, the adhesive strength of the NMC coating to the aluminum also increases. This effect occurs within a very short time after the first contact with air and complicates or hinders all recycling technologies that rely on separating the NMC coating from the aluminum carrier foil. The NMC obtained in this way also contains significant amounts of aluminum salts, which practically rules out its direct reuse as a positive electrode material and makes downstream processing steps necessary. This means that aluminum can also end up in processing steps that do not involve any further separation of aluminum, for example NMC separated by burning the binder. Even more serious are the effects of corrosion on the NMC itself. As shown, contact with the alkaline medium leads to destruction of the structure of the NMC, whereby it loses its electrochemical functionality and becomes unsuitable for functional recycling. A partial loss of the lithium contained in the NMC due to the penetrated moisture has an equally detrimental effect, even if no destruction of the structure has yet taken place. Last but not least, it should be considered that the alkaline dissolution of the aluminum carrier foil results in the simultaneous release of hydrogen^12,47^.

## Methods

### General procedure

Pouch cells with a capacity of 50 Ah, which contained NMC622 as active material, were used for the experiments. First, the discharged batteries were opened in an argon atmosphere. Half of the positive electrode foils from the battery pack were still separated under Argon atmosphere. Finally, the foils were stored under vacuum. The remaining foils were separated and stored in ambient air at a temperature of υ = 22 °C and a humidity of φ = 55%. After defined storage times (5, 30, 60, 120, 200, 260, 320, 1440 min) the positive electrode foils were decoated. Unless otherwise stated, functional recycling was simulated with a high-pressure water-jet for decoating the positive electrode foils. The positive electrode foils separated under argon atmosphere and stored under vacuum were exposed to ambient air the following day and also decoated in a time-dependent manner (10, 30, 75, 120, 180, 240, 300, 1440 min).

For the determination of aluminum content, the positive electrodes were stored in a terrarium where humidity was set at 55%. At the given times (60, 180, 300, 420, 540, 660, 780, 1650, 3300, 7350, 13,700 min), a foil was removed and part of the foil was treated at 550 °C for 3 h to thermally remove the binder. Then, the active material was removed from the aluminum foil and dissolved in hydrochloric acid for ICP-OES analysis.

In another series of experiments, the positive electrode foils, initially separated under argon atmosphere, were exposed to atmospheres with defined humidity. The humidity was adjusted using saturated salt solutions (φ = 22% CH_3_COOK; φ = 44% K_2_CO_3_; φ = 65% NH_4_NO_3_; φ = 80% (NH_4_)_2_SO_4_)^50^ by transferring a Petri dish filled with the salt solution and the positive electrode foil in a sealed vessel. The positive electrode foil, previously separated under Argon atmosphere, was positioned in this vessel and stored at 22 °C for a total of 18 h in the respective atmosphere.

### Confocal microscopic examination of the aluminum collector

The analysis of the surface of the positive electrode foils has been performed by confocal microscopy. A Sensofar PLμ neox 3D profiler (Sensofar) has been used for this purpose. The measurements were made with a 5 × (NA = 0.15) objective. The frame rate of the video is 1 min^-1^. Surface analysis was performed with a 20 × (NA = 0.4) objective. The surface ratio was calculated as described in ^44^.

After decoating the positive electrode foils in a simulated water jet process, the aluminum foils were examined with a 5 × objective (confocal microscope). The surface of the Al foils was recorded as a so-called extended image (6 images per foil, image 39 × 60 mm^2^). Since in this mode of operation the focal plane is not changed, the acquired image is an optical microscope image. Using the open source program "Gwyddion", the image was aligned (Plane Level), corrected for background (Remove Polynomial Background), and the holes created on the transparency were declared as grains (Mark Grains by Threshold), and the cumulative area of the holes (grains) relative to the total area was output as the measured value A_R_.

### SEM–EDX analyses

The analysis of the NMC surfaces were performed with a scanning electron microscope (Zeiss Evo MA15). Energy dispersive X-ray analysis (EDX) was used for spatially resolved elemental distribution. The secondary electron image as well as the elemental analysis has been recorded with a detector system from AMETEK® operating at 20 kV.

### ICP-OES analyses

Elemental analyses were performed by ICP-OES (iCap 6500 DUO, Thermo Scientific) using an HF-resistant sample introduction system (PTFE cyclone nebulizer chamber, Glass Expansion; MiraMist parallel path nebulizer, Burgener) and the following measurement conditions: RF incident power of 1150 W, an Argon plasma flow rate of 12 L min^−1^, an auxillary gas flow rate of 0.5 L min^−1^, a nebulizer Argon gas flow of 0.6 L min^−1^, and a sample uptake rate of 50 rpm.

The determination of lithium (Li), phosphorus (P) and sulphur (S) in the electrolyte was performed after aqueous dilution in radial plasma viewing mode at the following emission wavelengths. Li: 610.362 nm, 670.784 nm; P: 177.495 nm, 178.284 nm, 185.942 nm, 213.618 nm; S: 180.731 nm and 182.034 nm, respectively.

Aluminum in the positive electrode coating was determined after acid digestion in axial plasma viewing mode at emission wavelengths of 308.215 nm, 309.271 nm, 394.401 nm and 396.152 nm.

Quantification was performed in each case using matrix-matched multipoint calibrations prepared from single-element standards of 1 g L^−1^ (Sigma Aldrich).

### Ion-chromatographic analyses

The detection of fluoride in the electrolyte was carried out after ion chromatographic separation via conductivity detection after chemical suppression of the total conductivity (881 Compact IC pro, Deutsche Metrohm GmbH). Separation was carried out at 40 °C on a 250 × 4.0 mm ASupp5 column (Deutsche Metrohm GmbH) with an eluent composition of 1 mmol L^−1^ NaHCO_3_ and 3.2 mmol L^−1^ Na_2_CO_3_ and an eluent flow of 0.7 mL min^−1^.

### Raman confocal microscopy

Characterization of the NMC was performed using a confocal Raman microscope (DXR SmartRaman, Thermo Fisher Scientific). A 532 nm laser with a power of 2 mW was used as the excitation source. The laser beam has been focused on the NMC surface with a 50 × objective, resulting in a focused spot with a diameter of ≈ 0.8 μm, and a spot distance of 10 µm. The detector was arranged in the backscattering configuration and equipped with a grating with 900 grooves mm^−1^. All Raman spectra were recorded in the wavenumber range between 60 and 1800 cm^−1^.

### Laser-induced breakdown spectroscopy (LIBS)

LIBS measurements were performed using a CORALIS instrument (LTB Lasertechnik Berlin, Germany). LIBS experiments were carried out using 2…5 mJ laser pulses of a NdYAG laser at 1064 nm with a focal spot size of 20 µm. At each measuring point the signal was accumulated over 3 laser shots. The time delay between laser pulses and detection was set to 1 µs. The gain of the EM-CCD detector was set to 200. The spectral resolving power of the instrument is 15,000 λ/Δλ.

## Supplementary Information

Supplementary Video 1.

Supplementary Information 1.

Supplementary Information 2.

## Data Availability

The datasets used and/or analysed during the current study available from the corresponding author on reasonable request.
