# Supplementary material for: A phenomenological and quantitative view on the degradation of positive electrodes from spent lithium-ion batteries in humid atmosphere
Source: Sci Rep. 2023 Apr 6;13:5671. doi: 10.1038/s41598-023-32688-0 (PMC10079828; doi:10.1038/s41598-023-32688-0)
Supplement: Supplementary file 2 — Supplementary Information 1. [file 41598_2023_32688_MOESM2_ESM.pdf]

Supplementary Information

A Phenomenological and Quantitative View on the Degradation of Positive Electrodes from Spent Lithium-Ion Batteries in Humid Atmosphere

Thomas Langner[1*], Tim Sieber[1], Anja Rietig[1], Virginia Merk[2], Lutz Pfeifer[2] and Jörg Acker[1]

[1] Brandenburg University of Technology Cottbus – Senftenberg, Department of Physical Chemistry, D-01968 Senftenberg, Germany

[2] LTB Lasertechnik Berlin GmbH, D-12489 Berlin Germany

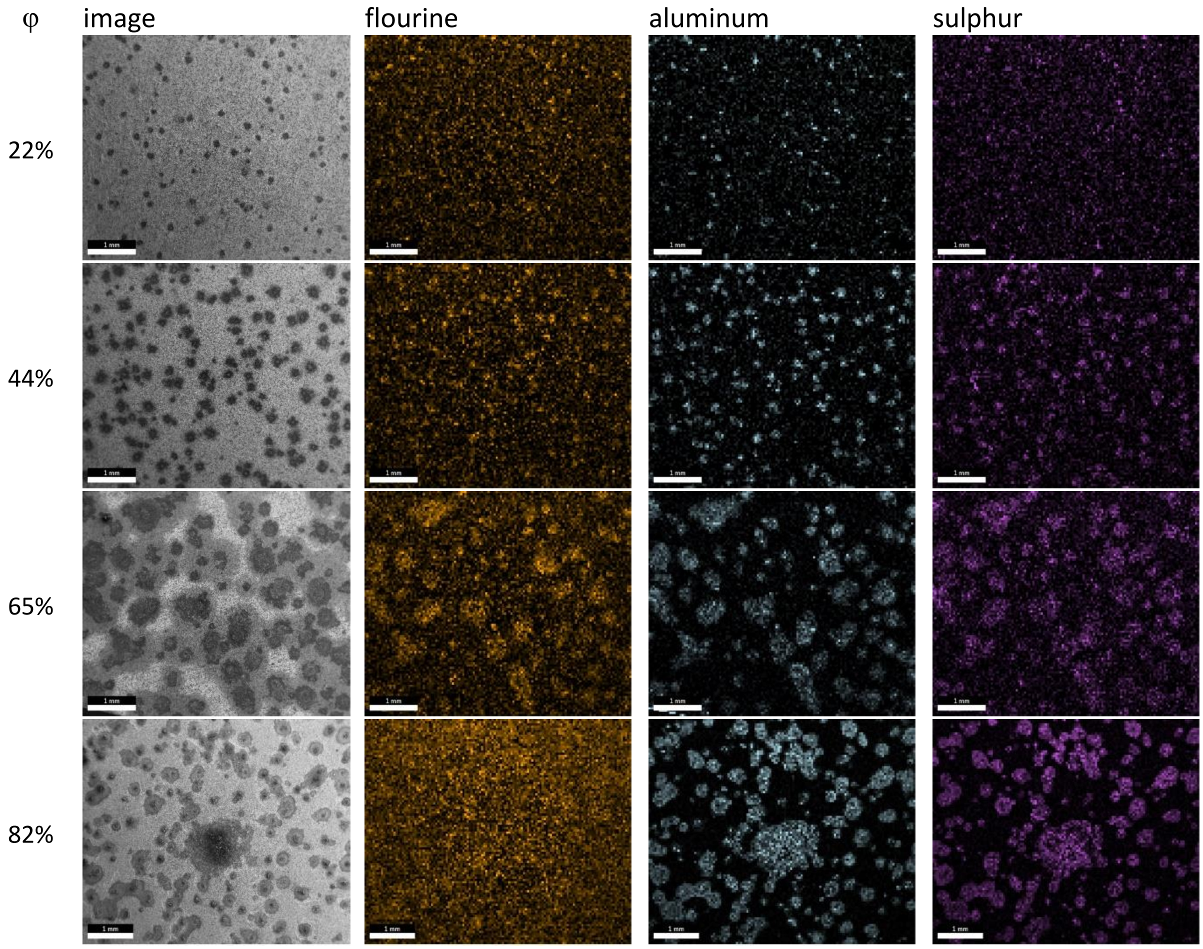

Supplementary Figure S1. SEM images and SEM-EDX analyses of a fixed section of 5.5 mm x 4 mm on the NMC coating with substrate contact area as a function of air humidity

Supplementary Table S1. SEM-EDX analysis of the Element contents (atom-%) on the substrate contact surface of the NMC coating with increasing air humidity

Element	22%	44%	65%	80%
C	19.0	15.2	20.76	18.24
O	24.7	22.4	22.66	25.68
F	2.72	2.88	4.57	3.21
Al	0.32	1.23	2.64	4.18
P	4.05	4.05	4.33	4.54
S	0.40	1.01	1.27	1.51
Mn	9.46	10.3	8.47	8.23
Fe	5.84	6.32	5.54	5.35
Co	8.45	9.21	7.48	7.44
Ni	25.10	27.39	22.28	21.61

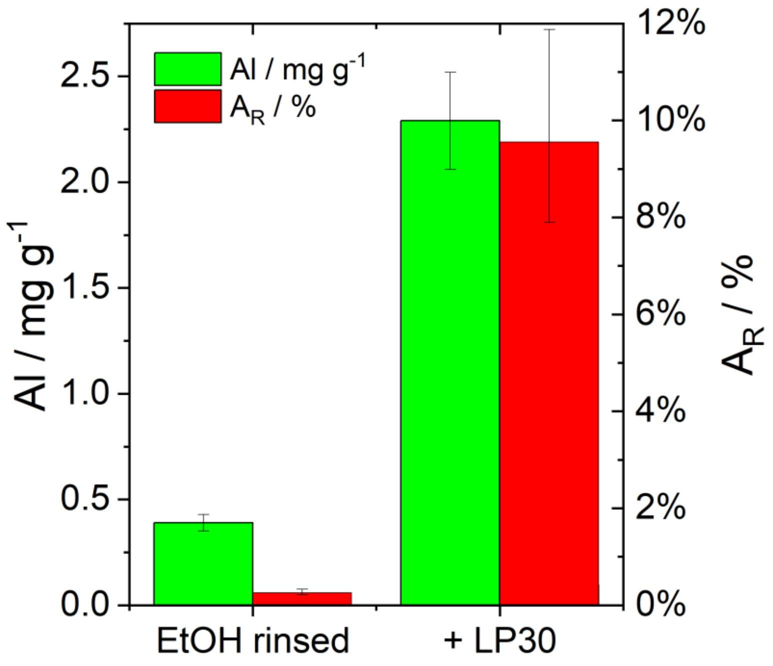

Supplementary Figure S2. Comparison of the aluminum content and the hole area (A_R) of a positive electrodes that was rinsed with Ethanol (EtOH rinsed) and an electrode that was rinsed in Ethanol and subsequently a LP30 electrolyte was added, positive electrodes were stored for 24 h at a humidity of $\varphi \approx 60\%$

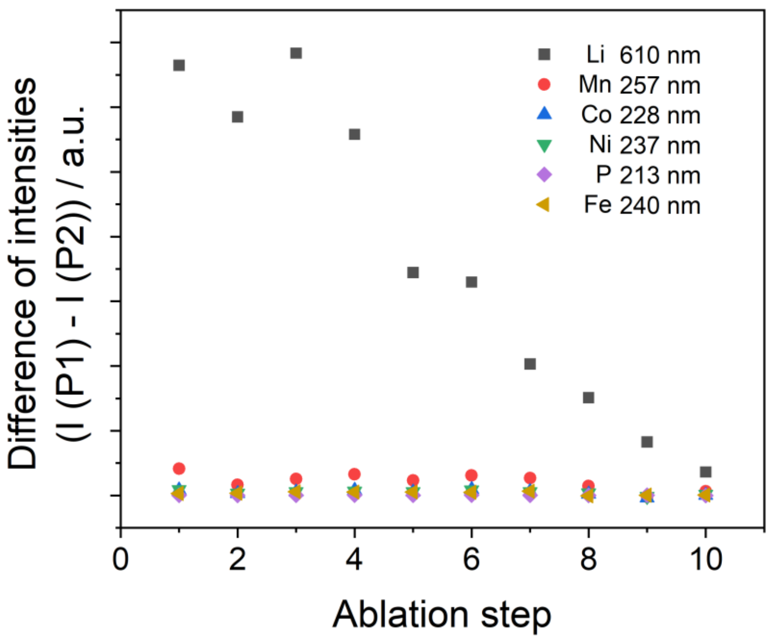

Supplementary Figure S3. difference in average emission intensities for each ablation step of the measurements of point 1 and point 2

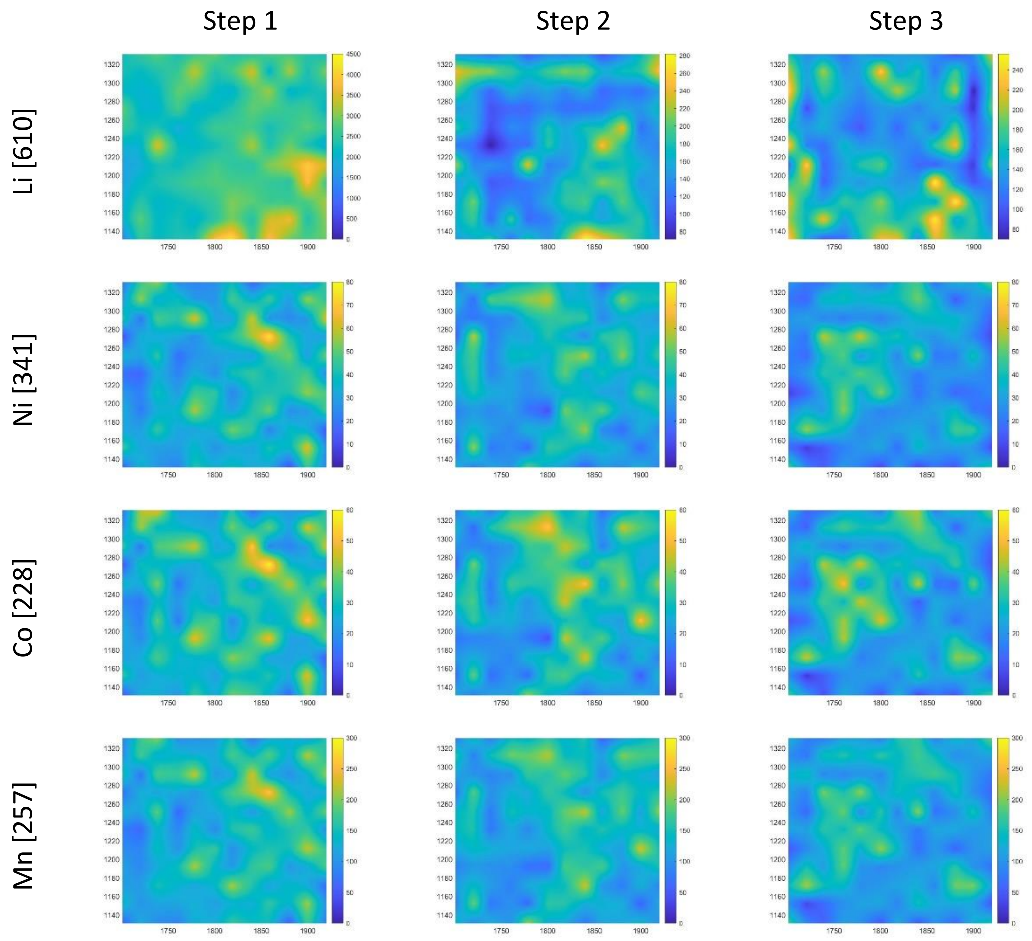

Supplementary Figure S4. LIBS analysis of a crater of the NMC backside for Lithium @ 610.36 nm, nickel @ 231.60 nm, cobalt @ 228.62 nm and manganese @ 257.61 nm, step means ablation step, size of mapped area 200 µm x 220 µm, the color bars display the color associated with the absolute intensity (I in cts.)

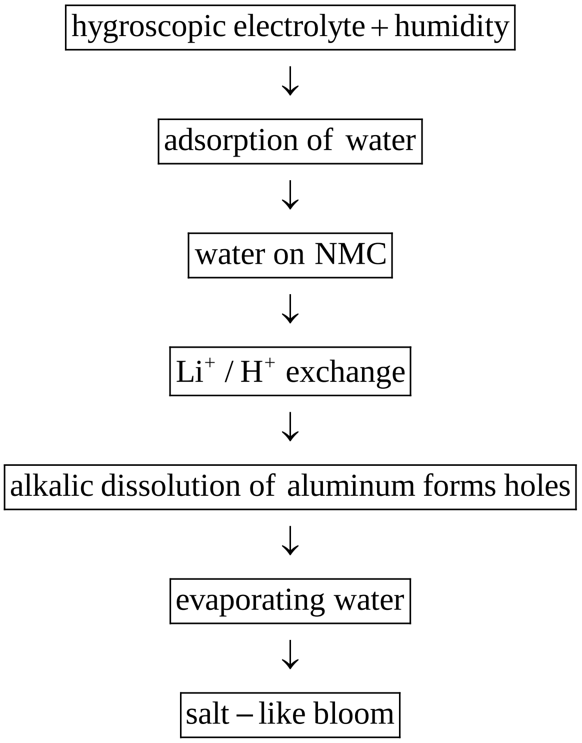

Supplementary Figure S5. Chemical reaction scheme for the corrosion process
