# Supplementary material for: A phenomenological and quantitative view on the degradation of positive electrodes from spent lithium-ion batteries in humid atmosphere
Source: Sci Rep. 2023 Apr 6;13:5671. doi: 10.1038/s41598-023-32688-0 (PMC10079828; doi:10.1038/s41598-023-32688-0)
Supplement: Supplementary file 3 — Supplementary Information 2. [file 41598_2023_32688_MOESM3_ESM.docx]

Supplementary Video S1. Time lapse of the accumulation of moisture on the surface of a positive electrode under a humidity of φ = 100% during a time frame of 100 min at a section 2.55 mm x 1.91 mm
